# Moderate delayed sowing increases grain number per spike by escaping low temperature stress and optimizing post-jointing temperature of winter wheat in Southwest China

**DOI:** 10.3389/fpls.2026.1899006

**Published:** 2026-07-17

**Authors:** Yong Ye, Zhenyu Liang, Tingting Zhu, Dahai He, Jiabo Chen, Xiulan Huang, Shimin Yang, Hongkun Yang, Gaoqiong Fan

**Affiliations:** 1 Crop Eco-physiology and Cultivation Key Laboratory of Sichuan Province, Sichuan Agricultural University, Chengdu, China; 2 Key Laboratory of Crop Eco-Physiology & Farming System in Southwest China, Ministry of Agriculture and Rural Affairs, Chengdu, China; 3 State Key Laboratory of Crop Gene Exploration and Utilization in Southwest China, Ministry of Science and Technology, Chengdu, China

**Keywords:** grain number per spike, low temperature, sowing date, wheat, yield

## Abstract

Global warming has increased the intensity and frequency of extreme low temperature events. Winter wheat in Southwest China lacks an overwintering dormancy period and undergoes an earlier and prolonged jointing stage, rendering it highly vulnerable to jointing-stage freezing injury. This study aimed to explore whether adjusting sowing date (SD) via delayed sowing could mitigate freezing injury at the jointing stage in Southwest China. A two-year field experiment was carried out with the winter wheat cultivar Shumai 1963 with five sowing dates, including early sowing (October 23, S1), normal sowing (October 30, S2), and late sowing (November 6, S3; November 13, S4; November 20, S5). The results showed that moderate sowing delay (7–14 days) prolonged the vegetative growth period and postpones the onset of jointing of wheat, thus effectively avoiding the risk of freezing injury. A 7-14-day sowing delay significantly improved grain yield by increasing grain number per spike (GNPS). Further analysis revealed that delayed sowing elevated the daily mean temperature (MT) from jointing to anthesis, reduced spikelet abortion and significantly increased fertile spikelets and grains across basal, central and apical spike positions. Benefiting from improved spike development, S3 and S4 raised grain yield by 4.0%-17.0% and significantly enhanced harvest index (HI) and effective accumulated temperature use efficiency (TUE) relative to normal sowing (S2). In conclusion, postponing sowing by 7–14 days is a feasible agronomic practice to evade spring low temperature, optimize post-jointing thermal environment and improve wheat productivity in Southwest China under climate warming.

## 1 Introduction

Wheat (*Triticum aestivum*) is one of the most widely cultivated cereal crops globally, supplying approximately 20% of total dietary calories and protein intake (Luo et al., 2023). As an indispensable staple crop, it underpins global food security and sustains rural livelihoods in major grain-producing regions. In recent decades, global warming has become a dominant climatic trend across the 21st century (Tierney et al., 2020), which has markedly raised the frequency and severity of extreme temperature events (Onyemaobi et al., 2022). These climatic perturbations have disturbed crop phenology in key wheat belts, hampering crop growth and yield formation and posing serious risks to global food security (Tao et al., 2022).

In China, low temperature stress has long been a primary constraint to wheat production, especially in the Southwestern wheat-growing region (Zhang et al., 2014). Such stress predominantly occurs during the jointing to heading stages, causing yield losses ranging from 15% to 60% (Wang et al., 2022a). Although climate warming has raised ambient temperatures across these areas, it also shortens the vegetative growth period and accelerates reproductive development of winter wheat, rendering the crop more prone to low temperature injury (Li et al., 2015). Jointing represents a critical transition point between vegetative and reproductive growth phases. Following this stage, wheat enters a phase of concurrent vegetative and reproductive development alongside active spikelet and floret differentiation, which makes crops more vulnerable to low temperature damage (Fang et al., 2024; Zhang et al., 2022). Consistent with this trend, recent studies have confirmed that low temperature damage risks for wheat have intensified across southern China despite warmer winter conditions (Jiang et al., 2022; Kong et al., 2023; Zhang et al., 2025), highlighting an urgent demand for practical and efficient strategies to mitigate low-temperature stress and stabilize wheat yield.

Among existing agronomic management practices, sowing date optimization is a cost-effective, environmentally friendly, and widely applicable approach for crop stress regulation (Fu et al., 2023). Moderate delayed sowing has been widely validated as a reliable field measure to evade spring low temperature stress in wheat (Xiao et al., 2018). Importantly, within a reasonable sowing window, moderate delayed sowing does not inevitably cause yield loss. Benefiting from elevated winter temperatures under climate change, winter wheat can still accumulate adequate pre-winter biomass and develop a robust tiller population to maintain stable productivity (Liu et al., 2019; Basheir et al., 2023). Furthermore, field evidence has demonstrated that a moderate ten-day delay in sowing can significantly enhance root vigor and reproductive-stage photosynthetic capacity, facilitating superior grain yield formation and improving final wheat productivity (Rasmussen and Thorup-Kristensen, 2016). Consequently, identifying the optimal sowing window is essential to coordinate wheat phenological development and low temperature stress adaptation, thereby sustaining stable grain production under changing climatic conditions.

Sowing date optimization enables crops to match their key growth periods with altered climatic conditions, which effectively converts the extra heat resources induced by climate warming into yield productivity and alleviates the adverse impacts of future climate change on wheat production (Abbass et al., 2022). Nowadays, nearly all previous studies investigating SD-mediated frost mitigation focused on winter wheat grown in Northern China, where wheat undergoes a long dormant overwintering period to develop cold resistance. These regional conclusions cannot be directly transferred to the Southwest wheat belt, a unique low-latitude zone with mild winters. Local wheat cultivars sustain continuous growth throughout winter without dormancy, resulting in an early and prolonged jointing stage that highly overlaps with spring freezing events. Quantitative linkages among sowing date, phenological staggering, reproductive-stage thermal conditions, spike fertility and final grain yield remain poorly quantified for this specific eco-climatic region. Therefore, this two-year field experiment was conducted to fill the above research gaps and provide quantitative, targeted agronomic guidance for frost risk management of rice-stubble winter wheat under climate warming in Southwest China.

## 2 Materials and methods

**Figure 1:**
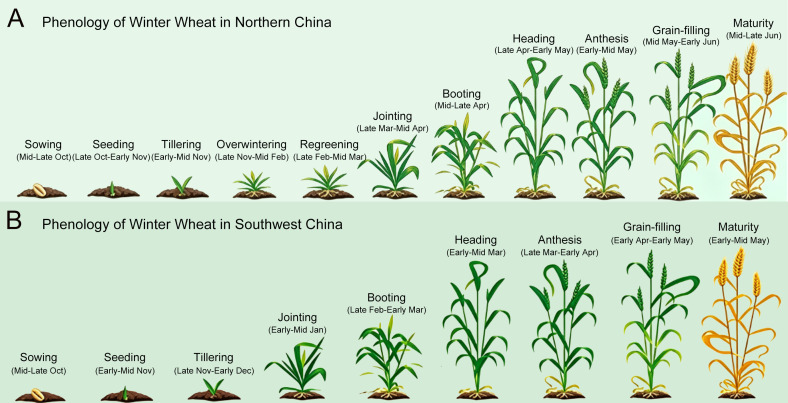
Comparative schematic diagram of winter wheat phenology in Northern and Southwest China. Growth periods of winter wheat in Northern China **(A)** feature distinct overwintering and regreening stages; the developmental sequence of Southwest China winter wheat **(B)** lacks dormancy phases, leading to early jointing and high frost risk. Parenthetical texts mark the standard ten-day occurrence period for each phenological stage. This phenological divergence creates entirely different frost response patterns to sowing date adjustment between the two wheat regions.

As China’s third major winter wheat-producing region, the Southwest wheat region possesses unique eco-climatic characteristics of low latitudes and warm winters. Distinct from Northern winter wheat, the local main spring wheat varieties lack obvious overwintering and regreening stages (**Figure 1**), which accelerates the whole development cycle. The jointing stage of Southwest wheat plants initiates early and lasts from mid-January to early March. This critical developmental window coincides with frequent low temperature freezing injury and overlaps the vulnerable period of young spike differentiation and floret primordia development. Such huge regional differences in frost risk and crop developmental rhythm mean SD regulation conclusions derived from Northern wheat belts cannot be directly applied locally, which forms the core ecological premise of this two-year field experiment.

### 2.1 Experimental design and management

**Figure 2:**
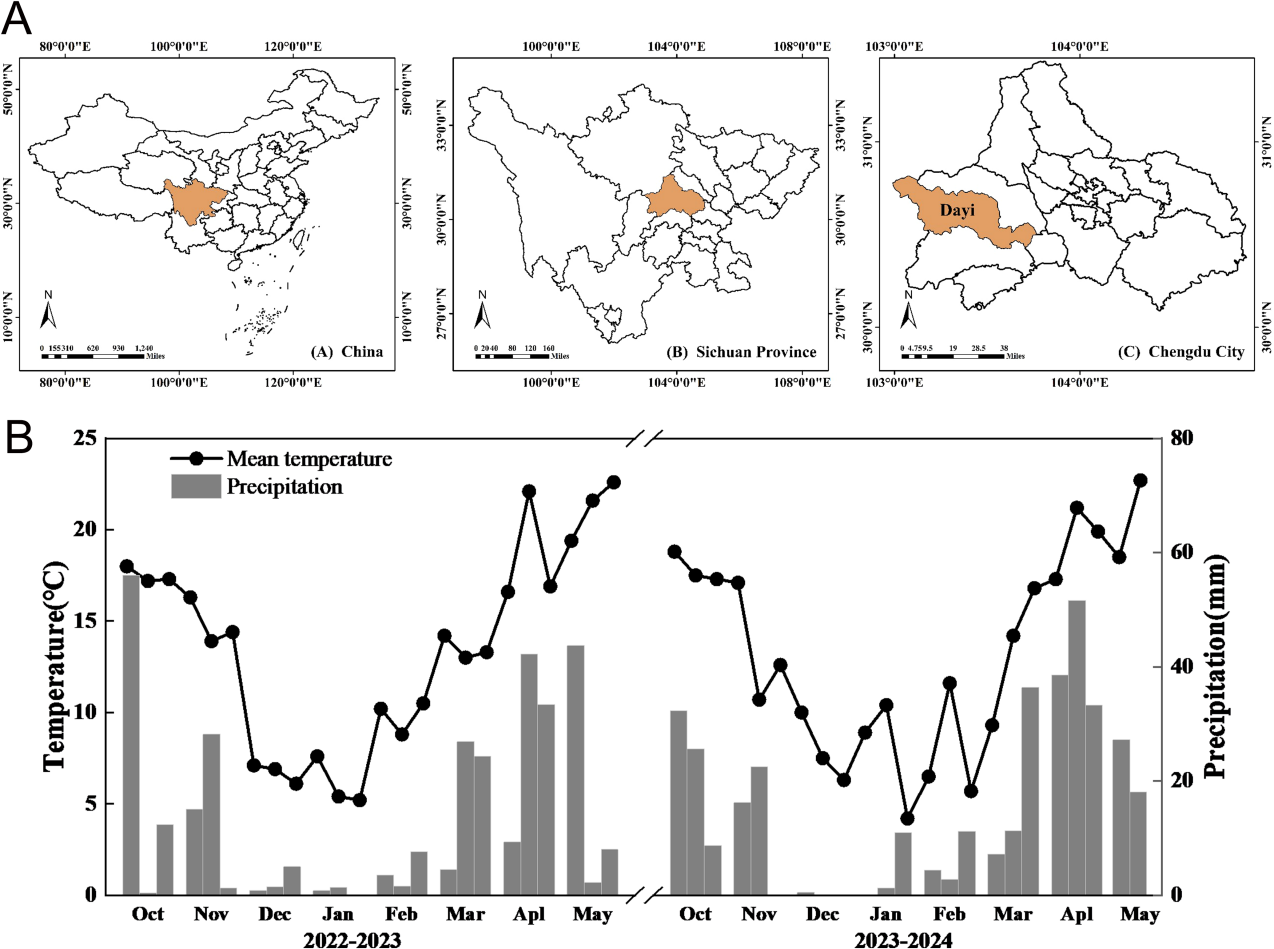
Location of the experimental site at Dayi County, Chengdu City, Sichuan Province, China **(A)**, and mean temperature and precipitation during two winter wheat growing seasons (2022–2023 and 2023-2024) **(B)**.

Field experiments were conducted across two successive wheat growing seasons from 2022 to 2024 in Dayi County, Chengdu City, Sichuan Province, China (30°58′ N, 103°53′ E; **Figure 2**). Rice was the preceding crop in the experimental field. Prior to sowing, soil samples were collected from the 0–20 cm topsoil layer to determine basic chemical properties. The soil contained 41.97 g kg^-1^ organic matter, 1.95 g kg^-1^ total nitrogen, 0.27 g kg^-1^ total phosphorus, 13.26 g kg^-1^ total potassium, 10.38 mg kg^-1^ available phosphorus, 228.47 mg kg^-1^ available potassium, and 223.32 mg kg^-1^ alkali-hydrolyzable nitrogen. Meteorological data recorded during the experimental periods are presented in **Figure 2**.

The winter wheat cultivar ShuMai 1963 was used as the test material. The experiment was arranged in a completely randomized design with three replications. Five sowing date treatments were set: 23 October (S1, early sowing), 30 October (S2, normal sowing), 6 November (S3, one-week delayed sowing), 13 November (S4, two-week delayed sowing), and 20 November (S5, three-week delayed sowing). Treatment S2 represented the local optimal sowing date for high yield, as verified by previous field trials (Liang et al., 2025). Each experimental plot covered an area of 12 m².

The fertilization ratio of N: P_2_O_5_: K_2_O was 2:1:1. The total application rates were 180 kg hm^-2^ for nitrogen, 60 kg hm^-2^ for P_2_O_5_ and 60 kg hm^-2^ for K_2_O. All phosphorus and potassium fertilizers were applied once as basal fertilizer. Nitrogen fertilizer was split into three applications: 60% as basal fertilizer, 30% as seedling fertilizer at the tillering stage, and the remaining 10% as topdressing at the jointing stage.

Seeds were sown by hole sowing after rotary tillage, with a row spacing of 20 cm and hill spacing of 10 cm. Seedlings were thinned to one plant per hill at the three-leaf stage. For weed management, the mixed herbicide containing florasulam and carfentrazone-ethyl (4% + 6% suspo-emulsion) was uniformly sprayed on leaf surfaces at a rate of 120 mL·hm^-2^ at the 3–5 leaf stage of wheat. All herbicide solutions were diluted with clean water at 450 L·hm^-2^ and applied using a knapsack sprayer under windless sunny conditions. For disease and pest protection, the spray mixture contained tebuconazole (43% SC) at 300 mL·hm^-2^, imidacloprid (20% WDG) at 225 g·hm^-2^, and potassium dihydrogen phosphate at 1.5 kg·hm^-2^. All agents were dissolved in clean water at 450 L·hm^-2^ and evenly sprayed onto wheat canopy with a knapsack sprayer to control rust, powdery mildew, aphids and supplement foliar potassium nutrition.

### 2.2 Data collection and measurements

The exact dates corresponding to the main reproductive periods of tillering (first tiller emerging from the leaf sheath), jointing (2 cm growth of the first internode at the base), anthesis, and maturity (95% of the seeds reaching the harvest standard) were accurately observed and recorded in the field for each treatment of wheat and the duration of each stage of its growth was calculated. Effective Accumulated Temperature (EAT) was calculated for each growth stage based on the daily mean temperature (MT), maximum temperature (T_max_), and minimum temperature (T_min_) of wheat during the growing season obtained from the local meteorological station at the study site concerning the method of (Huang et al., 2024), and the EAT was calculated using the following formula:


$$\text{EAT }\left(\text{°C}\right)\;=\sum \left(\text{MT}-{\text{T}}_{0}\right)\;\times \text{ days during wheat growth season},\text{ MT}>0\text{°C}$$

where T_0_ (wheat is 0 °C) is biological zero.

Field evaluations of wheat productivity were conducted annually at physiological maturity, with the number of effective spikes (FSN) per unit area determined in each plot. Random 1m^2^ wheat samples were harvested from three representative plots to assess grain yield and 1000-grain weight (TGW). Additionally, a total of 60 spikes were collected from each plot to measure grain number per spike (GNPS), spike length, the number of fertile spikelets, and the number of abortive spikelet, and the grain number grains per spikelet. The harvest index (HI) was calculated using hand-threshed samples. Final grain yields in each plot were quantified by manual harvesting, adjusted to 13% moisture content for uniformity across treatments.

### 2.3 Statistical analysis

All statistical analyses were performed using SPSS 27.0 software (IBM Corporation, Chicago, IL, USA). For *post-hoc* multiple comparisons, the Duncan’s multiple range test was adopted at the significance level of *P* < 0.05 to determine the statistical differences among treatment means. Data visualization and figure construction were conducted using OriginPro 2024b (OriginLab Corporation, Northampton, MA, USA) to present results in a clear and interpretable manner.

## 3 Results

### 3.1 Effects of sowing date on yield and yield components in winter wheat

Sowing date significantly altered winter wheat grain yield and its components across the two consecutive growing seasons (**Table 1**). During the 2022–2023 growing season, the FSN continuously decreased with delayed sowing. In contrast, GNPS increased gradually under moderate sowing delay, with the maximum values recorded in S3 and S4 treatments. The TGW fluctuated slightly across treatments, reaching its maximum in S5, while moderate delays (S3 and S4) maintained relatively stable TGW levels. For grain yield, moderate sowing delay (S3 and S4) resulted in optimal performance, which significantly higher than those under early sowing (S1), normal sowing (S2), and extreme late sowing (S5). Compared with normal sowing (S2), grain yield of S3 and S4 increased by 4.0% and 4.4%. During the 2023–2024 growing season, yield components responded to sowing date in an overall similar pattern (**Table 1**). The S2-S5 treatments had relatively stable FSN, with no significant differences detected among them. The GNPS increased steadily with delayed sowing, reaching a peak in S4, which was significantly higher than other treatments The TGW decreased continuously as sowing was postponed. The grain yield of S3 and S4 increased by 14.0% and 17.0%, compared with normal sowing (S2).

**Table 1:** Effects of sowing date on yield and its components of winter wheat during 2022–2023 and 2023–2024 growing seasons.

Year	Sowing date	FSN (×10^4^·hm^-2^)	GNPS	TGW (g)	Yield (kg·hm^-2^)	HI
2022-2023	23 October (S1)	480.42 ± 18.90 a	37.13 ± 0.32 c	49.19 ± 0.40 b	7878.45 ± 383.50 b	0.44 ± 0.02 d
	30 October (S2)	442.22 ± 8.88 b	41.45 ± 1.89 b	48.41 ± 0.03 cd	8122.59 ± 157.45 b	0.47 ± 0.01 c
	6 November (S3)	425.14 ± 9.65 bc	43.05 ± 0.38 b	48.68 ± 0.15 bc	8450.71 ± 54.29 a	0.48 ± 0.02 b
	13 November (S4)	417.36 ± 1.04 c	46.39 ± 0.15 a	47.98 ± 0.57 d	8476.39 ± 59.82 a	0.51 ± 0.05 a
	20 November (S5)	338.06 ± 4.86 d	36.98 ± 1.33 c	50.06 ± 0.28 a	5506.55 ± 85.33 c	0.49 ± 0.03 b
2023-2024	23 October (S1)	372.36 ± 0.80 b	22.62 ± 1.42 e	54.09 ± 0.37 a	4771.04 ± 150.02 c	0.30 ± 0.05 c
	30 October (S2)	415.23 ± 6.94 a	32.38 ± 0.74 d	52.41 ± 0.65 b	6510.43 ± 117.06 b	0.39 ± 0.01 b
	6 November (S3)	411.67 ± 10.44 a	38.64 ± 0.20 c	46.26 ± 0.88 c	7423.40 ± 207.22 a	0.48 ± 0.03 a
	13 November (S4)	404.27 ± 3.11 a	44.43 ± 0.29 a	42.75 ± 0.63 d	7618.75 ± 95.98 a	0.49 ± 0.07 a
	20 November (S5)	406.97 ± 7.11 a	42.31 ± 0.26 b	38.59 ± 0.27 e	7299.51 ± 347.64 a	0.47 ± 0.05 a

FSN, fertile spike number; GNPS, grain number per spike; TGW, 1000-grain weight. HI, harvest index. Different letters represent significant differences between treatments in the same growing season for each parameter (Duncan’s test, *P* < 0.05).

HI was markedly affected by sowing date across two growing seasons (**Table 1**). In 2022-2023, HI gradually rose from S1 to S4 and then declined at S5. The S4 treatment achieved the maximum HI (0.51), which was significantly higher than S1, S2 and S5. Compared with normal sowing (S2), HI of S3 and S4 increased by 2.1% and 8.5%, respectively. During the 2023–2024 growing season, HI exhibited a similar changing tendency, with S3 and S4 showing recording the highest HI, significantly exceeding early sowing (S1). Compared with S2, HI was improved by 23.1% (S3) and 25.6% (S4). Extremely delayed sowing (S5) slightly reduced HI relative to S3 and S4 in both years. Overall, appropriately postponed sowing (7–14 d, S3-S4) optimized source-sink relationship and significantly improved harvest index, which partly explained the yield promotion under moderate late sowing.

Over the two growing seasons, moderate sowing delay (7–14 days of delay) achieved a good balance among yield components. A substantial rise in GNPS compensated for the minor loss of FSN, leading to higher grain yield. Excessive sowing delay (S5) resulted in a sharp decline in FSN in 2022–2023 and a significant reduction in TGW in 2023-2024, limiting wheat yield potential. Collectively, an appropriate sowing window (S3 to S4, early to mid-November) optimized the coordination of yield components and maximized winter wheat yield, whereas both premature and extremely late sowing hampered yield formation.

### 3.2 Path coefficient analysisof yield components to grain yield

Path analysis was performed to separate the direct and indirect effects of three yield components on grain yield (**Table 2**). Across both growing seasons, GNPS exhibited the largest direct positive effect on yield, with direct path coefficients of 0.5881 in 2022–2023 and 0.8299 in 2023-2024. FSN showed a considerable direct positive contribution (0.6282) in 2022-2023, but its direct effect decreased (0.2505) in the warmer 2023–2024 season. The direct effect of TGW was relatively small and negative (-0.0501) in 2022–2023 and weakly positive (0.0554) in 2023-2024. The indirect effect of FSN via GNPS was positive and substantial, indicating a synergistic relationship between these two components. Combined-year path analysis further confirmed that GNPS was the dominant contributor to yield (0.8075), followed by FSN (0.4556). Total determination coefficients (R²) exceeded 0.95 in all analyses, indicating that the three yield components explained most of the yield variation. These results demonstrated that improving GNPS was the primary pathway for yield enhancement as delayed sowing, while FSN and TGW played secondary roles.

**Table 2:** Path coefficient analysis of yield components to grain yield during 2022–2023 and 2023–2024 growing seasons.

Year	Agronomic traits	Direct path coefficient	Indirect path coefficient		
			FSN	GNPS	TGW
2022-2023	FSN	0.6282	/	0.0678	0.0228
	GNPS	05881	0.0725	/	0.0406
	TGW	-0.0501	-0.2855	-0.4771	/
2023-2024	FSN	0.2505	/	0.5432	-0.0216
	GNPS	0.8299	0.1640	/	-0.0491
	TGW	0.0554	-0.0975	-0.7355	/
Combined years	FSN	0.4556	/	0.2053	-0.0194
	GNPS	0.8075	0.1412	/	-0.1089
	TGW	0.1618	-0.0547	-0.5436	/

FSN, fertile spike number; GNPS, grain number per spike; TGW, 1000-grain weight. “/” indicates no indirect path coefficient (diagonal terms).

### 3.3 Growth stage progression and temperature dynamics as affected by sowing date

**Figure 3:**
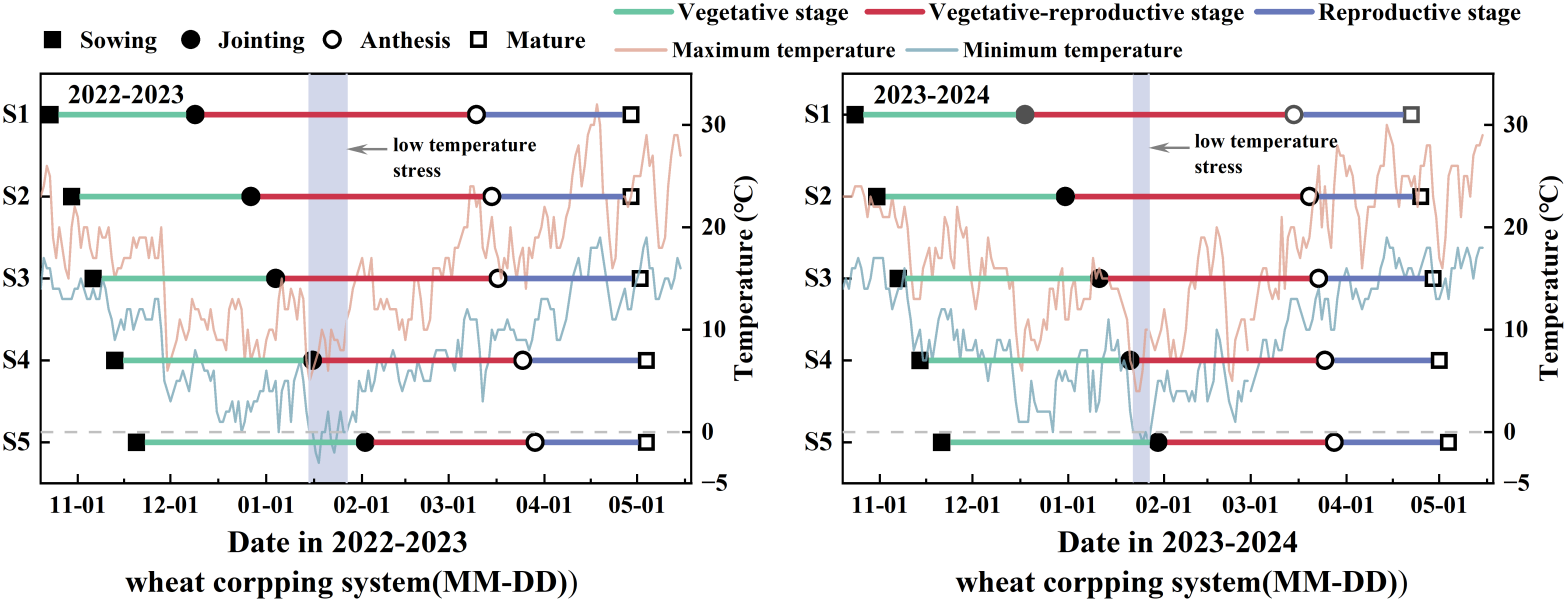
Dynamic changes of daily maximum and minimum air temperature and corresponding key phenological stages of winter wheat under five sowing-date treatments (S1-S5) during 2022–2023 and 2023–2024 growing seasons. The light blue shaded area represents the period of low temperature stress; green, red and blue solid lines denote vegetative, vegetative-reproductive and reproductive stages, respectively. Black solid square, solid circle, hollow circle and hollow square indicate sowing, jointing, anthesis and maturity dates, while salmon and light blue fluctuating curves stand for daily maximum and minimum temperature.

The timing and duration of key developmental stages of winter wheat were strongly influenced by sowing date across the 2022–2023 and 2023–2024 growing seasons (**Figure 3**). From early to late sowing (S1 to S5), the vegetative stage gradually increased, and the vegetative-reproductive and reproductive stage were progressively shortened. Specifically, a one-day delay in sowing prolonged the vegetative stage (from sowing to jointing) by 4 days but shortened the vegetative-reproductive transition stage (from jointing to anthesis) by 7 days, leading to a net reduction of 4.9 days in the total growth duration. Moreover, the key growth stages of winter wheat overlapped with low temperature stress to varying degrees across treatments. Freezing events were more severe and longer in duration during the 2022–2023 growing season than in 2023-2024, as shown by the blue shaded vertical bars (**Figure 3**). The vegetative-reproductive stage, a critical period determining the number of GNPS, is highly sensitive to freezing events. During the 2022–2023 growing season, the low temperature stress occurred in late January to early February, coinciding with the vegetative-reproductive stage of S1-S3. In 2023-2024, it occurred in late January, overlapping with the vegetative-reproductive stage of S1 and S2. These findings demonstrated that a 7-14−day delay in sowing effectively reduced losses in GNPS of winter wheat caused by low temperature stress during the vegetative−reproductive stage.

### 3.4 Effect of sowing date on spike characteristics of winter wheat

**Figure 4:**
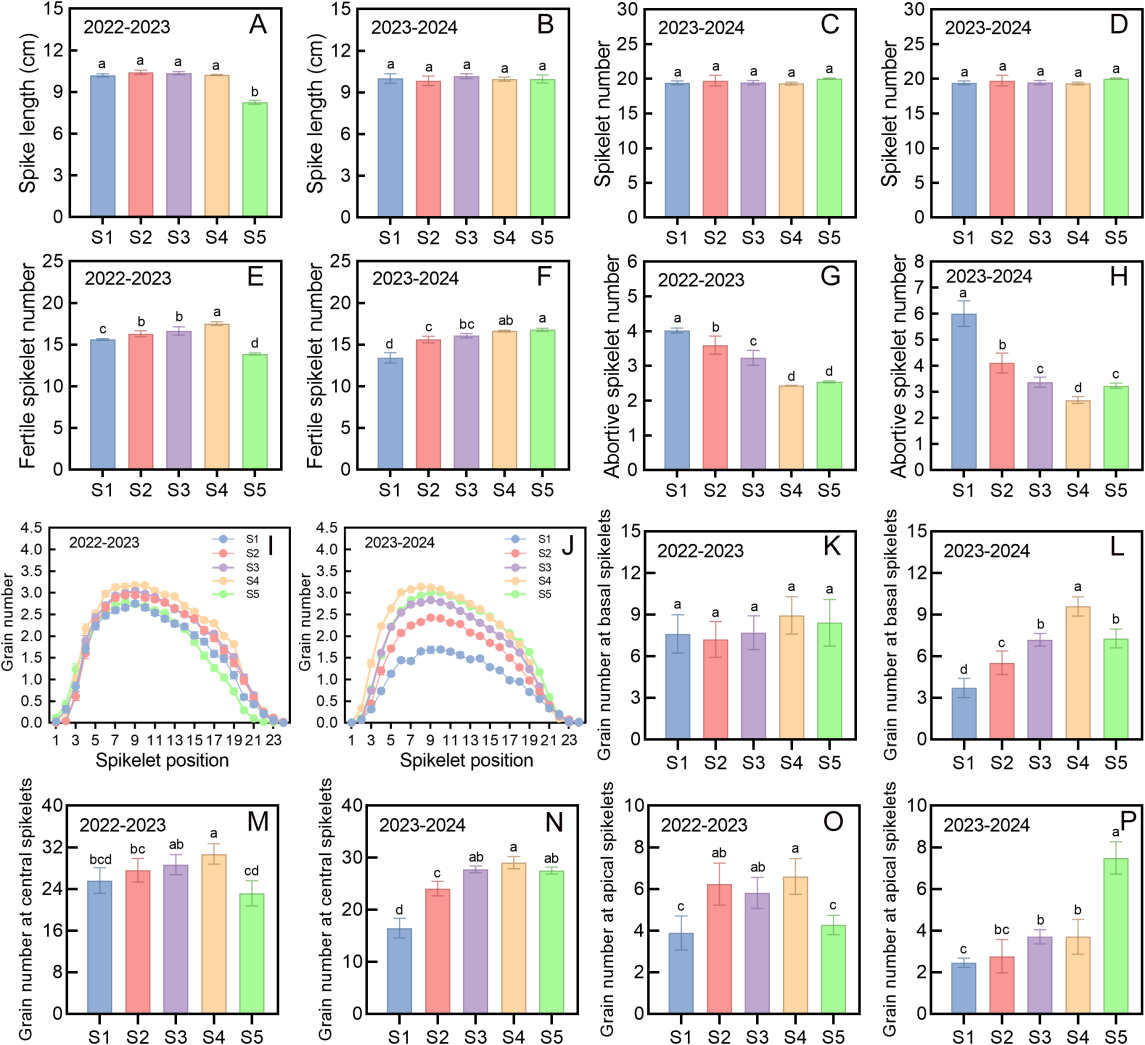
Effects of sowing date on spike length **(A, B)**, total spikelet number **(C, D)**, fertile spikelet number **(E, F)**, abortive spikelet number **(G, H)**, grain number at different spikelet position **(I, J)**, grain number at basal **(K–N)**, central **(K, N)** and apical spikelets **(O, P)** of winter wheat in 2022–2023 and 2023–2024 growing seasons. Different lowercase letters above columns denote significant differences at *P* < 0.05 according to Duncan’s multiple range test.

Sowing date significantly influenced spike morphological traits, including spike length, total spikelet count, fertile and abortive spikelet number, and positional grain number across basal, central and apical spikelets during the 2022–2023 and 2023–2024 growing seasons (**Figure 4**). Spike length and total spikelet number did not differ significantly among treatments S1 to S4 in both experimental years, while S5 markedly shortened spike length relative to earlier sowing treatments only in 2022–2023 (**Figures 4A–D**). Fertile spikelet number progressively increased from S1 to S4 and decreased at S5 across two seasons; S4 possessed the maximum fertile spikelets, which was statistically higher than S1 and S5 (**Figures 4E, F**). In contrast, abortive spikelet number was the highest under early sowing (S1) and gradually declined with postponed sowing until S4, followed by a slight rise at S5 in both seasons (**Figures 4G, H**).

Grain number displayed a unimodal pattern along rachis positions for all sowing dates, in which grain number per spikelet increased rapidly and peaked at central spikelets, then continuously decreased toward apical positions (**Figures 4I, J**). Remarkably, moderately delayed sowing (S3 and S4) substantially improved grain set across most spikelet positions compared with early (S1) or excessive late sowing (S5). For positional grain formation, grain number at basal spikelets remained similar among all sowing treatments in 2022-2023, whereas S4 generated significantly more basal grains than other treatments in 2023-2024 (**Figures 4K, L**). Central spikelets contributed dominantly to total grains per spike, with the maximum central grain number observed in S4 for both years. S5 caused sharp reduction of central grains in 2022–2023 and S1 always maintained the lowest value across two growing seasons (**Figures 4M, N**). Apical grain number steadily increased as sowing was postponed, peaking at S4 in both seasons, and S5 still sustained relatively high apical grain number especially in 2023-2024 (**Figures 4O, P**). In conclusion, appropriate delayed sowing (S3-S4) effectively reduced spikelet abortion and promoted grain development over the entire spike, whereas overly early or extreme late sowing restricted spike differentiation and grain setting of winter wheat.

### 3.5 Effective accumulated temperature use efficiency under different sowing dates

**Figure 5:**
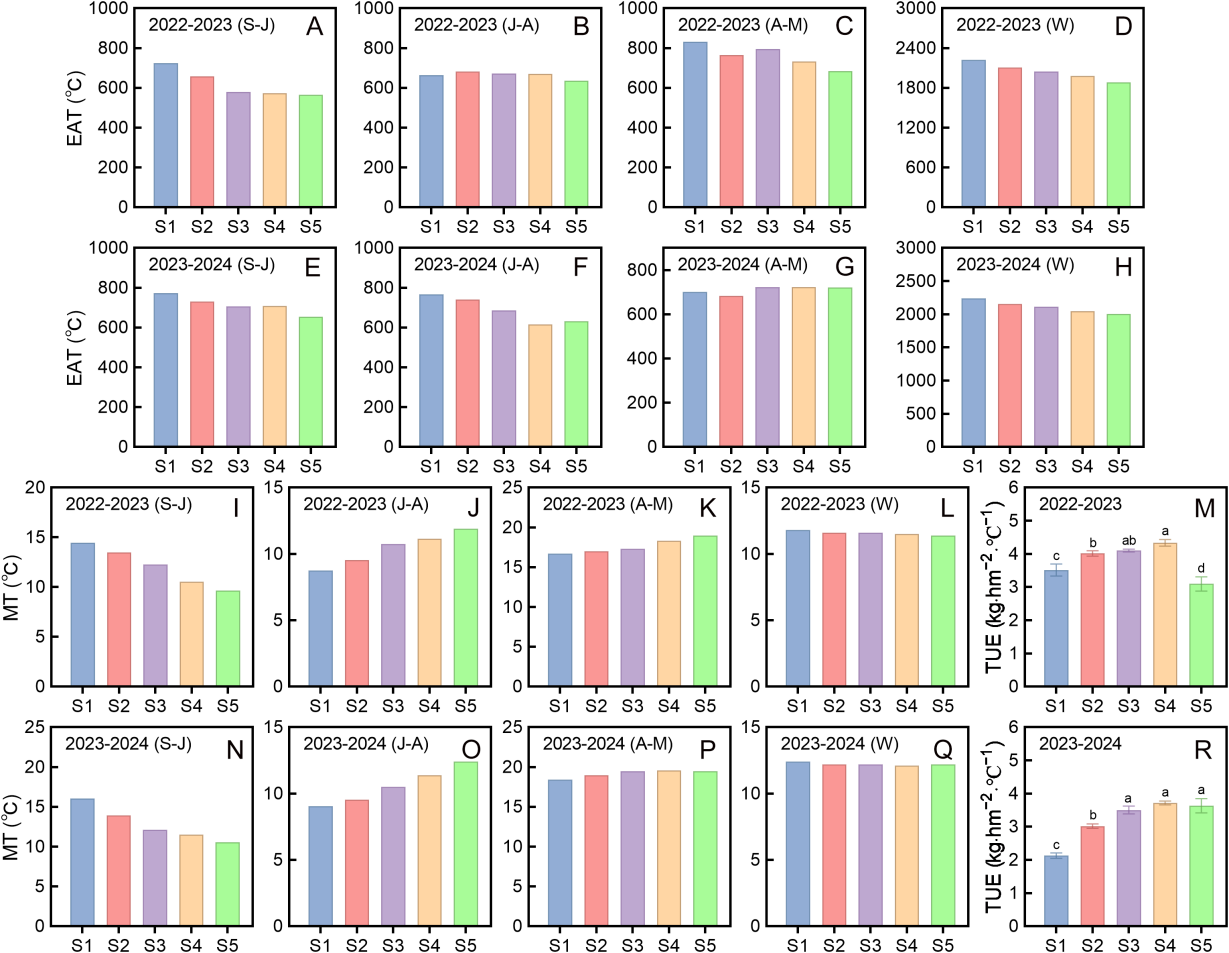
Variations in effective accumulated temperature [EAT, **(A–H)**], mean daily temperature [MT, **(I–Q)**] and effective accumulated temperature use efficiency [TUE, **(M, R)**] across different growth stages of winter wheat under five sowing-date treatments (S1-S5) during 2022–2023 and 2023–2024 growing seasons. S-J, sowing to jointing; J-A, jointing to anthesis; A-M, anthesis to maturity; W, whole growth period. Different lowercase letters above bars denote significant differences at *P* < 0.05 based on Duncan’s multiple range test. EAT, effective accumulated temperature; MT, daily mean temperature; TUE, effective accumulated temperature use efficiency (kg·hm^-2^·°C^-1^).

Sowing date significantly altered stage-specific EAT and MT across two growing seasons (**Figure 5**; **Table 3**). Pre-jointing EAT and MT gradually declined with postponed sowing in both years. From S1 to S5, pre-jointing MT decreased from 14.5 °C to 9.7 °C in 2022–2023 and from 16.0 °C to 10.6 °C in 2023-2024 (**Table 3**). By contrast, MT from jointing to anthesis continuously rose as sowing was delayed, increasing by 3.1 °C (2022-2023) and 3.4 °C (2023-2024) from S1 to S5. Post-anthesis MT increased steadily with delayed sowing in 2022–2023 while remained relatively stable among treatments in 2023-2024. The average MT over the entire growing season slightly declined with delayed seeding in both experimental years (**Figures 5L, Q**).

**Table 3:** Variations in effective accumulated temperature (EAT) and daily mean temperature (MT) across sowing to jointing, jointing to anthesis, anthesis to maturity and whole growth period under different sowing dates in 2022–2023 and 2023–2024.

Year	Sowing date	Sowing-Jointing		Jointing-Anthesis		Anthesis-Maturity		Whole growth period	
		EAT	MT	EAT	MT	EAT	MT	EAT	MT
2022-2023	S1	724.5	14.5	664.4	8.8	832.5	16.7	2221.4	11.8
	S2	658.4	13.5	681.5	9.6	765.7	17.0	2105.6	11.6
	S3	579.9	12.3	673.2	10.8	796.0	17.3	2049.1	11.6
	S4	574.3	10.5	671.1	11.2	733.3	18.3	1978.7	11.5
	S5	564.6	9.7	637.0	11.9	648.8	19.0	1850.4	11.4
2023-2024	S1	773.5	16.0	766.5	9.0	700.5	18.4	2240.5	12.4
	S2	730.5	13.9	741.5	9.6	683.5	19.0	2155.5	12.2
	S3	706.5	12.1	687.0	10.5	723.0	19.5	2116.5	12.2
	S4	708.5	11.5	616.5	11.4	723.5	19.6	2048.5	12.1
	S5	655.0	10.6	631.5	12.4	721.5	19.5	2008	12.2

TUE was significantly improved by moderate delayed sowing (**Figures 4M, R**). In 2022-2023, TUE gradually increased from S1 (3.55) to the peak value at S4 (4.28), followed by a sharp drop at S5 (2.98). For the warmer 2023–2024 growing season, TUE of early-sown S1 was only 2.13, while S3 and S4 achieved the highest TUE values of 3.51 and 3.72, respectively, and S5 maintained a relatively high TUE of 3.63. Collectively, appropriate 7–14 d postponed sowing (S3-S4) raised post-jointing MT, optimized spike differentiation and GNPS formation, and substantially elevated the thermal utilization efficiency of winter wheat in Southwest China.

### 3.6 Correlation analysis between thermal resources and yield components across key phenological stages under different sowing dates

**Figure 6:**
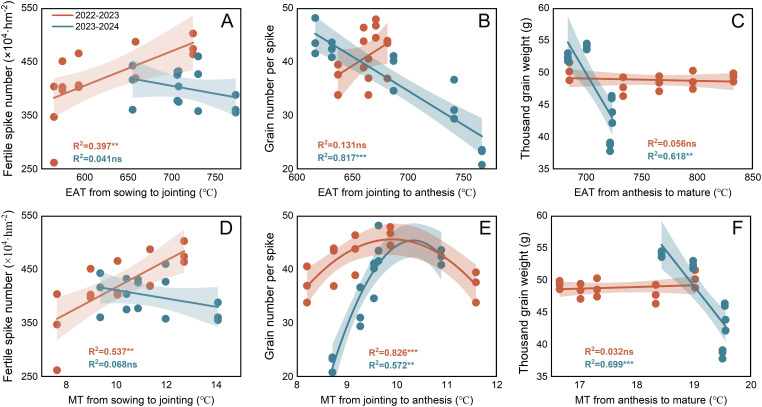
Regression relationships of fertile spike number **(A, D)**, grain number per spike **(B, E)** and thousand grain weight **(C, F)** with effective accumulated temperature (EAT) and mean daily temperature (MT) across three critical growth periods (sowing-jointing, jointing-anthesis and anthesis-maturity) in 2022-2023 (orange) and 2023-2024 (blue). Ns, non-significant at *P* > 0.05; ** and *** represent significance at *P* < 0.01 and *P* < 0.001, respectively.

EAT and MT at three critical growth intervals markedly influenced the formation of FSN, GNPS and TGW across the 2022–2023 and 2023–2024 growing seasons (**Figure 6**). Significant positive linear correlations were detected between pre-jointing EAT/MT and FSN in the 2022–2023 season, whereas no significant correlation was observed in the mild winter of 2023-2024 (**Figures 6A, D**). These findings suggest that sufficient pre-jointing thermal accumulation promotes effective spike differentiation in cropping years prone to low temperatures, while warmer winters alleviate such thermal constraints. GNPS responded non-linearly to MT from jointing to anthesis, following a unimodal quadratic curve that increased first and then decreased with rising MT in both experimental years (**Figure 6E**). The fitted curves revealed optimal MT thresholds around 9.9-10.3 °C for maximizing grains per spike, and the correlation reached extremely significant level for both seasons. Differently, linear correlation between jointing-anthesis EAT and GNPS was non-significant in 2022–2023 but significantly negative in 2023-2024 (**Figure 6B**). Post-anthesis thermal resources exerted divergent influences on TGW between years (**Figures 6C, F**). In 2022-2023, neither post-anthesis EAT nor MT was significantly correlated with TGW. By contrast, both EAT and MT after anthesis displayed significant negative linear relationships with TGW in 2023-2024, demonstrating that excessive post-anthesis temperature accelerated grain respiration consumption and restrained grain filling under delayed sowing.

## 4 Discussion

### 4.1 Suitable delayed sowing improves winter wheat yield via increasing GNPS in Southwest China

Wheat yield is co-determined by FSN, GNPS, and TGW, all of which are susceptible to shifts in SD. Most existing studies on SD regulation focused on Northern winter wheat zones with dormant overwintering, and their conclusions cannot be directly extrapolated to the low-latitude Southwest wheat belt (**Figure 1**). Here, two-year quantitative path analysis clearly identified GNPS as the dominant winter wheat yield-driving factor in Southwest China, with direct path coefficients reaching 0.5881 and 0.8299 across the two growing seasons (**Table 2**). Compared with conventional S2, suitable delayed sowing (S3-S4) increased grain yield by 4.0%-17.0% across two seasons, owing to the compensatory effect of increased GNPS offsetting partial FSN loss (**Table 1**). This compensation relationship between FSN and GNPS has been widely verified in previous wheat researches (Slafer et al., 2014; Zhu et al., 2019). Early sowing induced redundant ineffective tillers and aggravated intraspecific competition for photosynthate, limiting floret development and fertile spike formation, while moderate late sowing eliminated redundant tillers and allocated more assimilates to young spike development (Dai et al., 2017).

Moreover, moderately delayed sowing (S3 and S4) optimized source-sink allocation significantly raised harvest index, with HI rising by 2.1%-25.6% relative to normal sowing (S2) (**Table 1**). Extreme late sowing (S5) caused distinct yield limitation via sharply decreased FSN in cold year (2022-2023) and continuous TGW decline in warm year (2023-2024), which partially explains the adverse yield outcome of over-delayed seeding (Shah et al., 2020). Different from qualitative conclusions reported in Northern wheat systems (Liu et al., 2023a), our path analysis quantitatively confirmed that GNPS acted as the dominant yield-limiting factor, and the increased GNPS induced by moderate delayed sowing fully compensated for minor reductions in fertile spike number, forming a unique source-sink coordination pattern specific to Southwest wheat.

### 4.2 Moderate delayed sowing shifts wheat phenology to reduce the loss of GNPS caused by low temperature stress

The Southwest wheat region features an irreplaceable eco-climatic trait distinguishing it from Northern wheat belts: local cultivars sustain continuous vegetative growth throughout winter without dormant overwintering, leading to an early, prolonged jointing phase (mid-January–early March) that highly overlaps with spring freezing events, severely suppressing spike differentiation and grain formation (Xiao et al., 2018; Wang et al., 2022b). Northern wheat accumulates cold resistance during long winter dormancy, so frost risk mitigation via sowing adjustment operates via different physiological and phenological logic, making Northern management strategies inapplicable locally. This study supplied novel quantitative phenological evidence explaining how sowing delay evades low-temperature stress in Southwest China.

Our two-year results confirmed that one-day sowing delay extended the vegetative stage (sowing to jointing) by 4 days and shortened the freezing-sensitive jointing-anthesis phase by 7 days in Southwest China, achieving a net compression of total growth duration by 4.9 days. Such predictable phenological offset directly decouples young spike development from freezing exposure. In cold 2022-2023, low temperature coincided with jointing-anthesis of S1-S3, while S3 and S4 completely avoided cold injury. Similar avoidance effect was observed for S3 and S4 in the mild 2023–2024 growing season (**Figure 3**). Earlier-sown wheat suffered grain abortion once exposed to low temperature after jointing, consistent with Lin et al. (2023), who reported that cold stress during spike differentiation drastically reduces grain number at all spike positions. Lower MT under delayed sowing also enhanced seedling cold acclimation before stem elongation, further alleviating freezing vulnerability (Onyemaobi et al., 2022). Nevertheless, excessive three-week delay (S5) excessively compressed grain-filling duration and decreased TGW especially in warm winter (Farooq et al., 2011), revealing the upper limit of safe postponed sowing for local rice-stubble wheat. Unlike North China winter wheat with dormant period for cold tolerance (Liu et al., 2023b), phenology adjustment via sowing date plays a more prominent anti-freezing role in Southwest wheat production.

### 4.3 Moderate delayed sowing improves spike development and grain setting via elevating jointing-anthesis MT

Spike morphological differentiation and positional grain distribution were strongly modulated by altered MT from jointing to anthesis induced by sowing shift (**Figure 5**). MT gradually increased by 3.1 °C and 3.4 °C from S1 to S5 in two experimental years (**Table 3**), and such moderate temperature rise reduced spikelet abortion rate and increased fertile spike quantity, especially grains at basal and apical spikelets which were vulnerable to cold-induced abortion. A significant unimodal quadratic relationship existed between MT and GNPS, with optimal MT ranging from 9.9 to 10.3 °C for maximum grain set, matching the favorable temperature range (11.7 ± 1.61 °C) for spikelet development summarized by Farooq et al. (2011) and Zhou et al. (2020).

Delayed sowing rearranged the seasonal allocation of EAT and MT across key growth stages and remarkably improved whole-season TUE (**Figure 6**). Pre-joint EAT and MT declined gradually with postponed sowing, whereas reproductive-stage MT increased markedly, shifting redundant pre-winter heat toward spike differentiation period and converting surplus heat into grain yield (Abbass et al., 2022). S4 obtained the highest TUE (4.28 and 3.72 in two years), while early-sown S1 owned the lowest thermal utilization efficiency. Such thermal redistribution is the essential physiological basis for improved yield under moderate late sowing.

## 5 Conclusion

This two-year field experiment confirmed that moderate sowing postponement by 7–14 days (early to mid-November, S3-S4) serves as an efficient agronomic measure to mitigate low temperature stress and improve grain productivity in the Southwest wheat region, where wheat lacks dormant overwintering and faces severe spring freezing risk. Quantitatively, a 7–14 d sowing delay prolonged vegetative growth and postponed jointing timing, successfully staggering the freezing-sensitive young spike differentiation period away from spring low temperature stress. Meanwhile, delayed sowing elevated the MT during jointing to anthesis by 3.1-3.4 °C, which fell within the optimal thermal range (9.9-10.3 °C) for spike development, reduced spikelet and floret abortion, and significantly increased GNPS. Path analysis verified that improved GNPS constituted the dominant pathway to yield, which effectively offset slight losses in FSN and increased grain yield by 4.0%-17.0% alongside elevated HI. Furthermore, moderate delayed sowing optimized seasonal EAT distribution and substantially boosted TUE. Distinct from general sowing date strategies derived from Northern wheat zones, this study provides region-specific quantitative phenological, thermal and agronomic thresholds to support targeted freezing risk management for Southwest winter wheat.

## Data Availability

The original contributions presented in the study are included in the article/supplementary material. Further inquiries can be directed to the corresponding authors.
